# Beyond EGFR inhibition: multilateral combat strategies to stop the progression of head and neck cancer

**DOI:** 10.1038/s12276-018-0202-2

**Published:** 2019-01-16

**Authors:** Hyung Kwon Byeon, Minhee Ku, Jaemoon Yang

**Affiliations:** 1 Department of Otorhinolaryngology-Head and Neck Surgery, Soonchunhyang University College of Medicine, Seoul, Republic of Korea; 2 Systems Molecular Oncology for Head and Neck Cancer, Seoul, Republic of Korea; 3 Systems Molecular Radiology at Yonsei, Seoul, Republic of Korea; 4 Department of Radiology, Yonsei University College of Medicine, Seoul, Republic of Korea; 5 Research Institute of Radiological Science, Yonsei University, Seoul, Republic of Korea

**Keywords:** Cancer metabolism, Targeted therapies, Head and neck cancer, Cancer therapeutic resistance

## Abstract

Epidermal growth factor receptor (EGFR) overexpression is common in head and neck squamous cell carcinoma. Targeted therapy specifically directed towards EGFR has been an area of keen interest in head and neck cancer research, as EGFR is potentially an integration point for convergent signaling. Despite the latest advancements in cancer diagnostics and therapeutics against EGFR, the survival rates of patients with advanced head and neck cancer remain disappointing due to anti-EGFR resistance. This review article will discuss recent multilateral efforts to discover and validate actionable strategies that involve signaling pathways in heterogenous head and neck cancer and to overcome anti-EGFR resistance in the era of precision medicine. Particularly, this review will discuss in detail the issue of cancer metabolism, which has recently emerged as a novel mechanism by which head and neck cancer may be successfully controlled according to different perspectives.

## Cancer therapy: Combination strategies for head and neck cancer

South Korean researchers propose novel combination strategies for overcoming drug resistance and halting the progression of head and neck cancer (HNC). Although high levels of epidermal growth factor receptor (EGFR) protein in HNC correlate with reduced survival, patients’ response to the EGFR inhibitor cetuximab often declines rapidly after a short period of effectiveness. Hyung Kwon Byeon at Korea University College of Medicine in Seoul and colleagues review current knowledge of the mechanisms underlying cetuximab resistance. They suggest that evaluating a patient’s genetic profile and combining cetuximab with drugs that enhance the effects of inhibiting EGFR signaling pathways (with inhibitors of other EGFR family members or proteins that mediate EGFR entry to the cell nucleus, for example) as well as with agents that inhibit cancer cell metabolism could be a more effective approach for treating HNC.

## Introduction

Head and neck cancer (HNC) is the sixth most common cancer worldwide, as 40,000 new patients are diagnosed every year in the United States, and over 600,000 are diagnosed worldwide^1^. Despite the recent advancements in cancer diagnostics and therapeutics, the survival rates of patients with advanced HNC remain disappointing, and ~300,000 patients worldwide die from this disease every year. The anatomy of the head and neck is especially important since it is responsible for many vital functions such as respiration, phonation, and swallowing. Since locoregional invasion and metastases are relatively common and because esthetic or functional disabilities are inevitable following treatment, many difficulties are associated with the treatment of HNC. Conventional treatment modalities for HNC comprise surgery, chemotherapy, and radiotherapy. Although surgery still plays a definitive role in cases of resectable tumors, limitations in surgical resection clearly exist. Aggressive surgical resection itself would be most troublesome due to the complex and difficult anatomy, especially in cases of locally advanced tumors or recurrent tumors which have been treated with prior chemoradiotherapy. Chemotherapy and radiotherapy are routinely administered to HNC patients in primary definitive, adjuvant, or salvage treatment settings, but advanced cases are typically refractory. Therefore, novel treatment strategies are imperative for the management of HNC, especially in cases where the cancer has progressed beyond an initial stage of resection.

HNC has certain notable characteristics. For example, over 90% of all HNCs are pathologically squamous cell carcinomas, and 80–100% of HNCs feature epidermal growth factor receptor (EGFR) overexpression. Overexpression of EGFR is correlated with decreased survival, resistance to radiation, local treatment failure, and increased distant metastasis. Cetuximab, an EGFR monoclonal antibody, is the only FDA-approved targeted agent for HNC. However, treatment results were quite disappointing, unlike the initial expectations for this agent, as monotherapy responses were shown in only 10–30%, which suggests some form of intrinsic resistance. Moreover, patients who do achieve a clear tumor response eventually manifest disease progression due to acquired resistance to cetuximab. Numerous complex mechanisms underlie this treatment resistance. A low response rate to anti-EGFR targeted therapy, distinct inter- and intratumoral heterogeneity, relatively aggressive clinical features, and the functional and esthetic importance of head and neck anatomy are features that make HNC a challenging cancer to treat. This review article will discuss recent efforts in the discovery and validation of actionable targets in heterogenous HNC and methods to overcome anti-EGFR resistance in the era of precision medicine.

## The structure and biology of EGFR

EGFR is a 170 kDa transmembrane glycoprotein cell surface receptor that constitutes the ErbB/HER family, together with ErbB2 (HER2/neu), ErbB3 (HER3), and ErbB4 (HER4). All members of the HER family except for HER2 have known ligands. Six main ligands are known to bind to EGFR: EGF, heparin binding-EGF, TGF-α, amphiregulin, betacellulin, and epiregulin^2^. When EGFR binds to its ligand, it causes homodimerization or heterodimerization with other HER receptors (HER2, HER3) or other receptor tyrosine kinases (RTKs) such as MET or IGF-1 receptor. The activated EGFR affects four major signaling pathways: MAPK, PI3K/AKT/mTOR, PLCγ/PKC, and the JAK/STAT pathway^2^. Several studies have reported that some EGFRs exist as tetramers, which results in their inactivation, but the significance of this form has yet to be revealed^3,4^. EGFR can also act as a membrane-bound chaperone protein for the sodium-glucose cotransporter, SGLT1^5,6^. In HNC, known mutations in EGFR are rare, but the overexpression of EGFR together with one of its ligands, such as TGF-α, is relatively common. Autocrine or paracrine activation by EGFR ligands is important for EGFR activation in HNC. Tobacco smoke, a classic contributor to HNC can increase amphiregulin and TGF-α production, which results in direct EGFR activation. Another route of EGFR stimulation is by the indirect activation of G-protein-coupled receptors (GPCRs). GPCR ligands such as PGE2 or gastrin-releasing peptide (GRP) are increased in HNC, and consequent GPCR activation results in Src-mediated MMP activation; this causes the cleavage and release of EGFR proligands (TGF-α, amphiregulin), which ultimately leads to EGFR transactivation^2,7^. Furthermore, following EGFR activation, the expression of COX2 and its downstream product PGE2 is increased; PGE2 in turn transactivates EGFR, which establishes a positive feedback loop^2,8^.

**Fig. 1:**
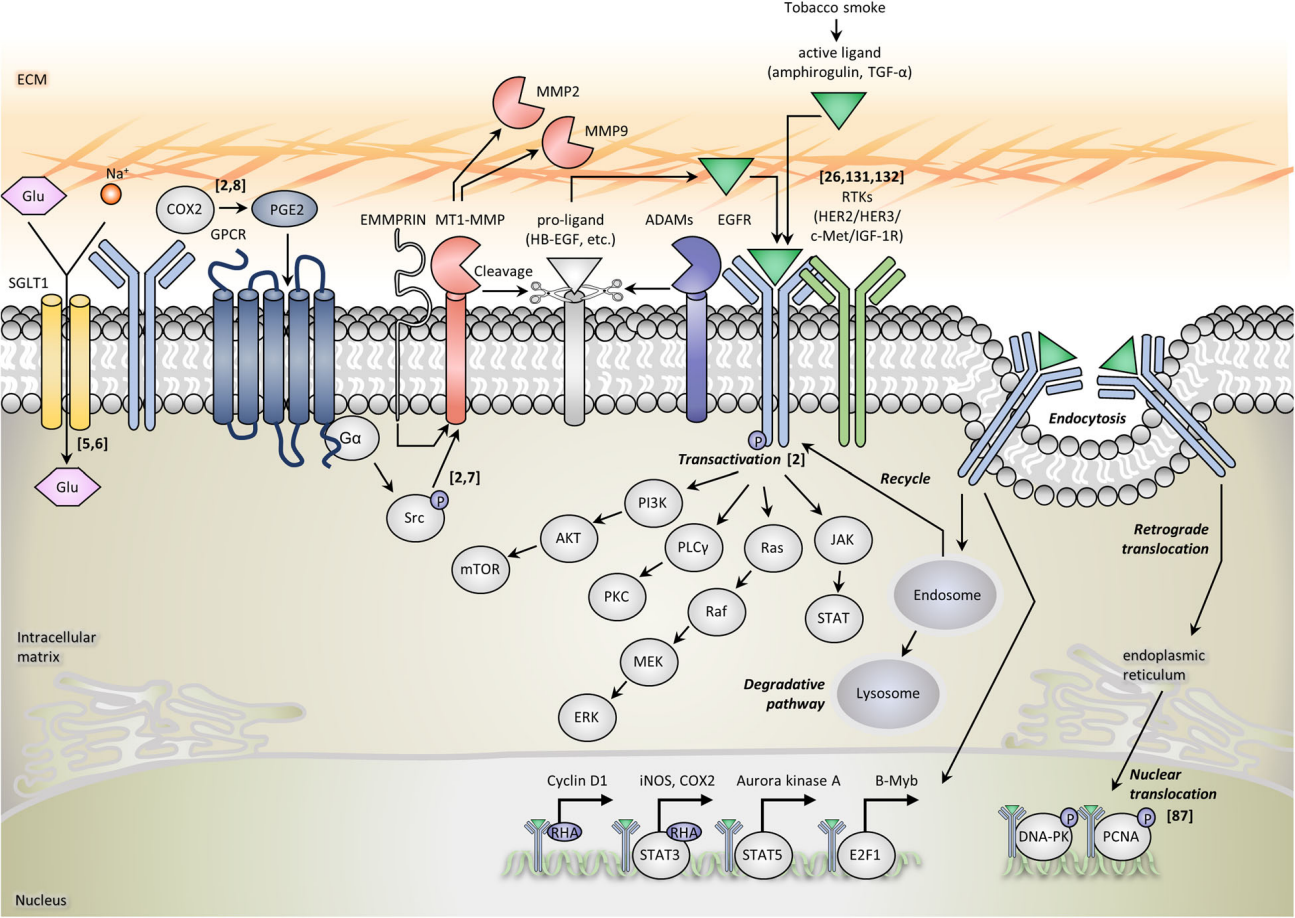
The biology of EGFR in head and neck cancer (HNC). Schematic diagram of the EGFR signaling network, its various interactions and mode of actions according to cellular localization. Numbers indicate relevant references in the text

The biological implications of EGFR are most important when EGFR is in its membrane-bound form as described above, where its activity is regulated by the quantity/quality of available receptors (overexpression or gain-of-function mutations in EGFR), interactions with other RTKs, and ligand availability. However, the EGFR signaling may also be spatially regulated by dynamic receptor cellular localization and recycling. EGFR itself can pose distinct signaling effects on different cellular compartments. Some have named these proteins ‘moonlighting proteins,’ where a single protein may have distinct functions according to its subcellular localization^9^. EGFR has several consequences within the cell once it has been engulfed during endocytosis. EGFR can enter the nucleus where it can serve several roles, be returned to the cell surface to continue its signaling function in its membrane-bound form, be directed to lysosomes for degradation, or remain active in endosomal compartments by mammary-derived growth factor inhibitor (MDG1), which leads to the activation of various downstream signals. Among these routes, the nuclear translocation of EGFR can play several important roles in anti-EGFR resistance^10^. Nuclear EGFR is known to be associated with poor survival, worse prognosis, and resistance to therapy. Several triggering mechanisms of EGFR nuclear translocation in HNC are known: EGFR ligands, cetuximab, EBV, radiation, and Src family kinase (SFK). Once in the nucleus, EGFR has two distinct functions. First, it acts as a transcription factor that binds to the promoters of multiple genes (iNOS, COX2, Aurora kinase A, B-myb, and cyclin D1) along with DNA-binding transcription cofactors (STAT3, STAT5, and E2F1). Second, nuclear EGFR can directly cause PCNA and DNA-PK phosphorylation due to its intrinsic tyrosine kinase activity. This increased EGFR activity can induce cell proliferation and promote the repair of DNA damage caused by chemoradiotherapy, which results in therapeutic resistance and ultimately cancer progression (Fig. 1).

## Tumors with EGFR overexpression and their characteristics

Overexpression or upregulated activity of EGFR is an important molecular characteristic that has been noted in numerous epithelial solid tumors such as colorectal cancer (CRC), non-small cell lung cancer (NSCLC), HNC, pancreatic cancer, breast cancer, and brain cancer. However, distinguishable mechanisms exist within these EGFR-overexpressing tumors. For instance, *EGFR* amplification and dysregulated EGFR expression together with *KRAS* mutations are commonly found in CRC whereas *EGFR-*activating mutations are important characteristics in NSCLC. Of the diverse somatic mutations in EGFR in NSCLC, exon 19 deletion and L858R mutation of the EGFR kinase domain are the most common forms, as they account for 85% of all *EGFR* mutations^11^. In HNC, however, EGFR overexpression is more commonly observed with rare events of *EGFR* mutations or *EGFR* amplifications. EGFR overexpression in HNC is also observed in normal tissue adjacent to the cancer, which supports the notion of field cancerization^12^. In short, EGFR functions more as a driver oncogene in NSCLC, while EGFR plays a role as the component of one of the many pathways that contribute to tumor growth in CRC and HNC.

## Approaches to EGFR inhibition in cancer

Two main classes of inhibitors target EGFR: monoclonal antibody (mAb)-based drugs and small molecule tyrosine kinase inhibitors (TKIs). The main action of mAbs is to bind to the extracellular domain (ECD) of EGFR, which blocks ligand-receptor binding and consequently results in the abrogation of EGFR dimerization. The mAb-receptor complex is then internalized after which it is consequently degraded, ultimately resulting in the downregulation of EGFR overexpression. The most well-known anti-EGFR mAb is cetuximab (chimeric mouse-human IgG1 antibody), which is the only FDA-approved targeted agent for HNC, but other agents such as panitumumab (fully humanized IgG2 antibody) are also under intense evaluation in HNC-based clinical trials^13,14^. In contrast the primary site of action of TKIs is within the intracellular tyrosine kinase domain of EGFR, where they compete with ATP to eliminate EGFR downstream signaling. TKIs are usually short-acting drugs since they tend to have a much shorter half-life than mAbs. TKIs have several advantages over mAbs such as oral administration and fewer hypersensitivity reactions. Reversible acting EGFR TKIs such as gefitinib and erlotinib have not shown a clinical benefit in HNC, but multitarget TKIs such as lapatinib (reversible dual EGFR and HER2 TKI), afatinib and dacomitinib (both irreversible EGFR, HER2, and HER4 pan-HER TKIs) have shown promise in various clinical trials^15–18^.

## EGFR-targeted mAbs

Anti-EGFR mAbs are generally used in cases of CRC and HNC. However, despite the overexpression of EGFR in these cancers, the initial response rates to cetuximab monotherapy are far from encouraging, and furthermore, treatment responses rapidly decline after a short period of effect. Generally, targeted drug resistance can be divided into the following two types: primary (intrinsic) and secondary (acquired) resistance. Naturally, resistance mechanisms vary among different cancers and the type of EGFR-directed agents used.

The major resistance mechanisms to EGFR-targeted mAbs that have been identified thus far are summarized in Table 1. In CRC in particular, the activation of a bypass signaling pathway, also referred to as ‘oncogenic shift,’ is a major mechanism of resistance to cetuximab. *KRAS* activation is an important mechanism of innate and acquired drug resistance, but resistance may also be mediated through other signaling networks such as MET, HER2/3, BRAF, and PIK3CA, which share the same mechanisms in other cancers. Additionally, in CRC, some have reported an acquired EGFR mutation in the ECD region (S492R), which hinders cetuximab binding. Unlike the oncogenic addiction of *EGFR*-mutant NSCLC, EGFR, as one of many pathways that contributes to tumor growth in CRC, leads to certain clinical implications. Treatment responses to EGFR inhibitor monotherapy will be relatively less pronounced and overcoming EGFR resistance may be less feasible due to alternate crosstalk mechanisms in CRC. Therefore, a combinatorial treatment strategy may be more applicable in CRC compared with NSCLC in which a single driver oncogene is responsible. These specific considerations in CRC have similar implications in HNC, which will be discussed in more detail below.

**Table 1:** Resistance mechanisms to anti-EGFR monoclonal antibodies

Major mechanisms	Action	References
Overexpressions of EGFR/ligands	Overexpressions of EGFR and TGF-α	24,112
Dysregulation of EGFR internalization and degradation by ubiquitination	EGFR is downregulated but its affinity to other activating signals are strengthened	24,113
MDG1 binding	MDG1-bound intracellular EGFR avoids extracellular targeting	114
Nuclear translocation of EGFR	Transcription of multiple genes or directly phosphorylates PCNA and DNA-PK	87
Enhanced SFK-mediated signaling	Promotion of EGFR nuclear translocation	113,115
EGFRvIII	Constitutively activated EGFR in a ligand-independent manner	116
KRAS mutation	Constant activation of EGFR downstream signals	117
PTEN loss	PI3K/AKT signal activation	117
Increased heterodimerization of EGFR or HER2 with HER3	PI3K/AKT pathway signal enhanced	24,115
Crosstalks	Crosstalk with HGF-MET	24,37
	Crosstalk with VEGF-VEGFR1	118,119
EMT	Local invasion and distant metastasis	120

## EGFR-targeted TKIs

EGFR TKIs are more commonly applied in NSCLCs, which exhibit oncogene addiction to EGFR signaling. The most common EGFR-activating mutation, L858R, is considered a predictor of sensitivity to EGFR TKIs. As with mAbs, EGFR-targeted TKIs also manifest various resistance mechanisms (Table 2). The most common mechanism of TKI resistance in NSCLC is the *EGFR* T790M ‘gatekeeper’ mutation, which is found in nearly 60% of patients who present with acquired resistance. This secondary kinase mutation results in a drug-resistant state of the cancer, where the actions of EGFR inhibitors are abrogated while its intrinsic EGFR kinase activity is maintained; this in turn contributes to ‘oncogenic drift’. This acquired resistance to first-generation EGFR TKIs such as erlotinib and gefitinib led to the clinical development of second-generation EGFR TKIs^19^. Second-generation TKIs such as afatinib and dacomitinib were designed specifically to enhance the treatment efficacy via the formation of irreversible covalent attachments to the EGFR kinase domain and action against a broader range of targets such as other HER family receptors (HER2, HER4) and structurally similar receptors (VEGFR). Their stronger binding activity to this secondary *EGFR* mutation revealed relatively more robust EGFR targeting ability, but these drugs are still limited. Therefore, third-generation TKIs were developed to specifically act against the T790M *EGFR* mutation. Osimertinib (AZD9291) has been recently approved by the FDA for NSCLCs harboring the *EGFR* T790M mutation^20^. Its primary mode of action is irreversible binding to EGFR with the T790M-mutation, but its effects against *EGFR* with a L858R mutation or an exon 19 deletion have also been demonstrated. However, a new form of tertiary *EGFR* C797S mutation has recently emerged, and ways to overcome resistance conferred by this mutation are currently being investigated^21–23^.

**Table 2:** Resistance mechanisms to anti-EGFR tyrosine kinase inhibitors

Major mechanisms	Action	References
EGFR mutations	T790M, C797S mutations	21, 121,122
EGFRvIII	Constitutively activated EGFR in a ligand-independent manner	123
PTEN mutation/loss	PI3K/AKT signal activation	124,125
KRAS mutations	Constant activation of EGFR downstream signals	1 26
Crosstalk	Increased expressions of HER2/HER3	2 6
	ADAM17 mediated NRG1 release leading to autocrine activation of HER2/HER3	127
	Crosstalk with MET	128
	HGF overexpression	129
	Crosstalk with AXL	41
	Crosstalk with VEGF-VEGFR	119,130
IGF-1R activation	Crosstalk, upregulation of IGF-1R	131
	Decreased expressions of regulators of IGF-1R ligands (IGFBP3/IGFBP4) leading to increased availability of IGF-1/IGF-2	132
EMT	Local invasion and distant metastasis	120
Histologic transformation	NSCLC to small cell lung cancer	133,134

## EGFR inhibitor resistance in HNC

**Fig. 2:**
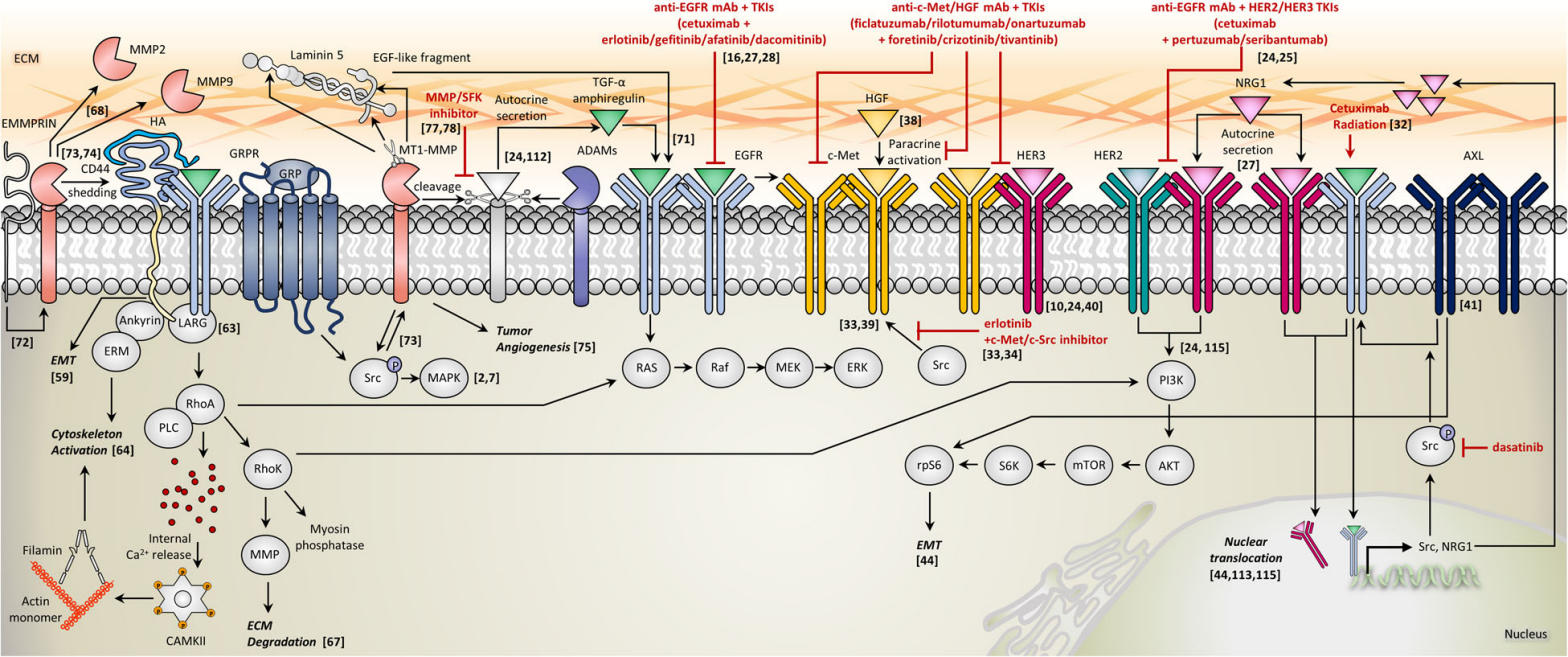
Major resistance mechanisms against EGFR inhibition in HNC. This schematic diagram illustrates reported resistance mechanisms to anti-EGFR monoclonal antibodies (mAbs) or tyrosine kinase inhibitors (TKIs) that are relevant in HNC. Inhibitors of specific targets are highlighted in red. Numbers indicate relevant references in the text

In HNC, most research has focused mainly on the underlying mechanisms of cetuximab resistance since it is the only FDA-approved targeted agent that is currently used in the clinic. From elucidation of the resistance mechanisms, many strategies to overcome such resistance have been be proposed (Fig. 2).

## HER3

Additional activation of HER3 signaling has been elucidated as one of the major, prominent mechanisms that underlies acquired resistance to cetuximab in HNC^10,24,25^. Upregulated HER3 signaling has also been recognized as a resistance mechanism to the EGFR TKI, gefitinib^26^. Phosphorylated HER3 in turn mediates potent activation of PI3K/AKT signaling. The activity of HER3 is dependent on EGFR and HER2, and HER2/HER3 heterodimerization is the main form contributing to cetuximab resistance in HNC. Therefore, simultaneous blocking of EGFR with either HER2 or HER3 has exhibited promising antitumor effects and has been proposed as an important strategy to overcome resistance to cetuximab. Combinatorial treatment of cetuximab with pertuzumab (2C4; HER2 monoclonal antibody)^24^ or seribantumab (MM-121; HER3 monoclonal antibody)^25^ results in effective blocking of both EGFR and HER3 signals and potent tumor suppression. Likewise, it has been demonstrated that lapatinib can effectively disrupt HER3 activation by blocking HER2/HER3 heterodimerization, either as monotherapy in intrinsically NRG1/HER3-enriched HNC^27^ or as a combinatorial treatment with cetuximab in cetuximab-resistant HNC (authors’ unpublished data). Pan-HER TKIs such as dacomitinib have also shown superior treatment efficacies compared with cetuximab or erlotinib alone in HNC cell lines^28^. Furthermore, the EGFR- and HER3-bispecific monoclonal antibody duligotuzumab (MEHD7945A) has recently been developed and holds promise^29^. Due to the high expression of NRG1 in HER3-enriched HNC, a significant role of NRG1-mediated autocrine signaling has been suggested in HER3-mediated cetuximab resistance. Therefore, inhibition with NRG1-neutralizing antibodies could be a potential treatment strategy^30,31^.

## SFKs

The SFKs are another important mediator of resistance to EGFR-targeted therapy in HNC. Generally, the SFKs are involved in anti-EGFR resistance and progression of HNC via three primary mechanisms. The first mechanism of EGFR resistance by SFKs is the mediation of cetuximab- or radiation-induced EGFR nuclear translocation, which leads to cetuximab resistance^32^. The blockade of SFKs by dasatinib treatment abrogates the process of EGFR nuclear translocation promoted by cetuximab or radiation. The second mode of action is that SFKs mediate the cleavage of EGFR proligands and consequent EGFR hyperactivation^7^. More specifically, Src is activated by the GRP/GRP receptor and contributes to the cleavage and extracellular release of TGF-α and amphiregulin, a process that is mediated by MMPs; this leads to EGFR and downstream MAPK activation^2,7^. The proteolytic release of TGF-α and amphiregulin by GRP stimulation is blocked not only by a MMP inhibitor but also by a SFK inhibitor. The third role of SFKs concerns ligand-independent activation of MET^33,34^. Notably, this mode of resistance is specifically relevant to erlotinib and not cetuximab^33^. In one study, Src inhibition resulted in MET inhibition^34^. Therefore, the addition of a MET or Src inhibitor to erlotinib treatment may lead to a synergistic effect in erlotinib-resistant HNC.

## HGF/MET

MET is involved in another well-established resistance mechanism of EGFR inhibition. HGF is the sole known ligand of MET. Genomic data of HNC reveals that gene amplifications or mutations in the *MET* gene are relatively rare, with ~20% of HNC presenting either an amplification or copy number gain and fewer than 1% harboring a gene mutation. However, HGF (50%)/MET (80%) overexpression is relatively common in HNC^35,36^. Compensatory activation of MET is the key mechanism that contributes to acquired resistance to cetuximab^24,37^. HGF acts mainly as a paracrine factor rather than as an autocrine activator of MET in HNC, and because it is secreted by cancer-associated fibroblasts, it is abundant in the tumor microenvironment^38^. Although this paracrine effect of HGF is the primary activating mode of MET, it can also be activated in a ligand-independent manner through the mediation of Src, particularly in erlotinib- or gefitinib-resistant tumors^33,39^. Furthermore, MET can also be activated to some degree by heterodimerization with HER3^10,24,40^, and therefore, blocking MET would be an important strategy to overcome resistance to anti-EGFR therapies. Three approaches have been established to target MET: anti-MET or anti-HGF mAbs such as ficlatuzumab, rilotumumab, and onartuzumab; TKIs such as foretinib, crizotinib, tivantinib, cabozantinib; a NK4 decoy, which is a truncated, soluble MET receptor that acts as an HGF antagonist.

## AXL

Together with Tyro-3 and MerTK, AXL constitutes the TAM family of RTKs. Previously, the oncogenic RTK AXL was implicated in resistance to EGFR TKIs such as erlotinib in HNC^41^, but AXL was also found to be both overexpressed and hyperactivated in cetuximab-resistant HNC^42^. More importantly, the elimination of HER2 or HER3 receptors in cetuximab-resistant HNC cells has no effect on EGFR phosphorylation, whereas AXL knockdown causes a prominent decrease in EGFR activity and significant inhibition of tumor proliferation. From these findings, additionally targeting the AXL appears to be a rational approach to overcome EGFR resistance, since HER2/HER3 signaling inhibition is not sufficient for complete tumor suppression^42,43^. Furthermore, AXL promotes EGFR nuclear translocation and transcriptional induction of NRG1 and SFKs, which leads to autocrine activation of HER3, EGFR-HER3 interaction, and EGFR activation^44^. This provides compelling evidence that the previously described major resistance mechanisms to EGFR inhibitors are all intimately connected to one another. AXL has further roles in the activation of rapamycin/ribosomal protein S6 signaling and the induction of epithelial to mesenchymal transition (EMT), which mediates resistance to PI3K and EGFR inhibition^44^.

## p53

The p53 protein is a tumor suppressor that plays a vital role in the suppression of cancer progression by promoting cell-cycle arrest, apoptosis, and senescence. The *TP53* gene is the most commonly mutated gene in HNC^45,46^, and loss of p53 function is found in more than 90% of HNC cases^35,47^. *TP53* mutations in HNC are correlated with poor clinical outcomes^48^, and p53 protein also plays a significant role in acquired resistance to EGFR inhibitors based on the identification of a robust loss of p53 in HNC cells that are resistant to cetuximab or erlotinib^49^. Furthermore, the loss of p53 also demonstrates cross-resistance to radiation, and therefore, restoration of p53 function resensitizes HNC to cetuximab and radiation, just as dasatinib enhances both cetuximab therapy and radiotherapy^32^. p53 regulates sensitivity to EGFR inhibitors by controlling EGFR downstream pathways such as ERK signaling and the PI3K/AKT pathway^50,51^. Targeting the cell cycle, including p53, therefore seems to be a promising therapeutic strategy in HNC.

The function of p53 is also important in cancer metabolism because it modulates glycolysis in several ways. p53 inhibits glycolysis by reducing the gene expression of *GLUT1* and increasing gene expressions of *TP53*-induced glycolysis and apoptosis regulator and phosphatase and tensin homolog deleted from chromosome 10^52,53^. In addition, p53 inhibits the pentose phosphate pathway, which is involved in nucleotide biosynthesis^54^. Furthermore, p53 induces the expression of synthesis of cytochrome c oxidase deficient homolog 2 and glutaminase 2 in the mitochondria for oxidative phosphorylation (OXPHOS)^55,56^. In cancer cells with functional loss of p53, the Warburg effect will therefore be accentuated, and thus targeting the p53 protein will have further important implications in HNC.

## EMT

For the progression and dissemination of cancer, tumor cells need to migrate, invade, and metastasize, as well as proliferate. Clinically, the invasiveness of a tumor is directly related to patient prognosis^57^. It is widely accepted that these processes are executed by EMT induction. During EMT, tumor cells at the primary site lose cell–cell contacts, engage in cytoskeletal remodeling, acquire mesenchymal and stem cell signatures, and display migratory phenotypes. As stated above, EMT is another mechanism that contributes to resistance to anti-EGFR therapies in HNC^58^.

In particular, expression of a stemness marker CD44 is increased during EMT^59^, and plays significant roles in HNC progression. The exons of the *CD44* gene are alternatively spliced to produce multiple variant isoforms of CD44 (CD44v), and of these, the CD44 isoforms v3, v6, and v10 are particularly significant in HNC^60^. The expression of these variant isoforms in HNC were found to be related to lymph node/distant metastasis, advanced disease, poor survival, and chemoresistance, and generally, CD44 expression is primarily concentrated at the invasive fronts of tumors^60,61^. CD44 has a principle role in the mediation of resistance to drug therapy including EGFR-targeted agents^61,62^. Hyaluronan (HA), which is a major constituent of the extracellular matrix (ECM), is the primary ligand of CD44. As HA binds to the CD44 receptor, which is localized at the cell surface, a CD44-EGFR complex is formed. This complex in turn initiates various downstream signals mediated by leukemia-associated Rho-guanine (LARG) nucleotide exchange factor. The HA/CD44-EGFR-LARG complex can activate Ras-mediated MAPK signaling or RhoA-mediated RhoK signaling, which leads to either myosin light chain phosphatase and MMP activation and consequent ECM degradation, or PI3K signaling activation^63^. Moreover, HA/CD44 signaling can induce cytoskeleton activation, either by RhoA/PLC-mediated intracellular Ca^2+^ release and subsequent calcium/calmodulin-dependent protein kinase type II activation, or by direct interaction of ankyrin and ezrin-radixin-moesin proteins with CD44^64^. The role of CD44 in metastasis has also been rigorously investigated in a recent report^65^. In that study, a subpopulation of CD44^high^ oral carcinoma cells was slow-cycling, exhibited overexpression of genes related to fatty acid metabolism, and was involved in lymphatic/distant metastasis rather than tumor proliferation. Furthermore, these specific cancer cells exhibited CD36 overexpression, a cell surface receptor which uptakes extracellular lipid to obtain ATP energy through lipid β-oxidation^66^. The contribution of CD44 to CD36-mediated fatty acid oxidation (FAO) in ‘initiating metastasis’ suggests a novel, alternative strategy of FAO suppression in HNC.

The interaction of tumor cells with the ECM is an integral factor in local invasion and metastasis of cancer. ECM degradation is caused by proteolytic enzymes, typically MMPs, that are secreted by tumor cells. MMPs are classified into collagenases (MMP-1, -8, and -13), gelatinases (MMP-2 and -9), stromelysins (MMP-3, -10, -11, and -27), matrilysins (MMP-7 and -26), enamelysin (MMP-20), metalloelastase (MMP-12), membrane-type MMPs (MMP-14 to 17, -24, and -25), and others (MMP-19, -21, -23, and -28) by dependent substrates^67^. In particular, MT1-MMP (MMP-14) is a zinc-dependent proteinase expressed in the cell membrane that is involved in the promotion of tumor growth and metastasis^68^. EGFR is activated by the cleavage of activating growth factor molecules or by the dispersion of receptor ligands within the ECM onto the cell surface^69^. Several proteinases such as MMPs and ADAMs regulate growth factors^70^. Specifically, MT1-MMP is involved in the dispersion of EGF ligands such as HB-EGF and EGF-like fragment released from the γ2 chain of laminin 5, which activate EGFR^71^. The activated EGFR signals in turn induce MT1-MMP expression and promote invasion of HNC by EMMPRIN-mediated MMP-2 and MMP-9 expression^72^. MT1-MMP also promotes intracellular signaling through Src and MAPK, and the shedding of CD44^73,74^. It was also reported that MT1-MMP modulates tumor-induced angiogenesis^75^. More noteworthy is that MT1-MMP supports the maintenance of energy metabolism via the mediation of the direct or indirect uptake of glucose and lipoproteins^76^. In short, MT1-MMP plays many key roles in growth, invasion, metastasis, and energy metabolism of cancer cells. Therefore, EGFR signaling and downstream gene expression could be regulated by a broad spectrum of MMP inhibitors such as clinically tested batimastat (BB-94), ilomastat (GM6001) and marimastat (BB-2516) or by the MT1-MMP-specific inhibitor NSC405020^77,78^.

## Therapeutic strategies beyond simple EGFR inhibition in HNC

### Reinforcement of oncogenic signaling inhibition

Although many important mechanisms contribute to resistance to EGFR-targeted therapies in HNC, EGFR is still a significant therapeutic target. The importance of EGFR overexpression aside, EGFR is an integral point for convergent signaling pathways, and EGFR targeting should form the basis of oncogenic signaling inhibition. Therefore, multilateral strategies that strengthen the inhibition of oncogenic signals should be employed.

Simultaneous inhibition of both the ECD and the intracellular tyrosine kinase domain of EGFR by combination of a mAb and a TKI can be a rational approach to enhance EGFR inhibition. The complementary actions of a mAb and TKI can be combined to present a deadly blow to the tumor by throwing a ‘HER1-2 punch’^79^. The synergistic effect of combining cetuximab with erlotinib or gefitinib have been extensively studied in many EGFR-dependent cancers^80–82^. One study showed a marked increase in EGFR mRNA in erlotinib-resistant tumors, which could be abrogated by cetuximab treatment^81^. Therefore, both EGFR downregulation and suppression of EGFR activity can be achieved. This dual inhibition of EGFR was also shown to be effective in HNC, where gefitinib or erlotinib still retained its antitumor activity in cetuximab-resistant cells^83^.

In consideration of crosstalk mechanisms with other HER family receptors following EGFR inhibition, the inhibition of a multitude of HER receptors would be an effective treatment strategy to overcome resistance. This so-called ‘horizontal targeting’ strategy in combination with EGFR inhibitors has been intensively investigated in NSCLC and has shown promising outcomes in both preclinical and clinical studies^84–86^. The combination of afatinib and cetuximab exerted a synergistic effect in EGFR TKI-resistant NSCLC from not only the inhibitory actions of multi-HER receptors but also from the dual inhibition of the extracellular and intracellular domains of EGFR^85^. Furthermore, this combinatorial strategy even showed promising results when administered as a first-line therapy in TKI-naïve NSCLC^86^. Multi-HER inhibitors such as lapatinib, afatinib, and dacomitinib have also shown encouraging results as single agents in HNC^16,27,28^, and combinatorial treatment of afatinib and cetuximab has shown promise in HNC^16^. For all the reasons discussed above, the combined treatment of cetuximab with either afatinib or dacomitinib deserves attention as an active area of investigation. Additionally, horizontal targeting of another major resistance molecule, MET could also be considered.

In distinction to the horizontal targeting strategy, vertical targeting of EGFR can be achieved by combinatorial inhibition of EGFR and RTKs such as SFKs or AXL, which mediate the nuclear translocation of EGFR^87^. This inhibition would be particularly advantageous since they are also involved in signaling crosstalk with molecules such as MET and HER3, which play mechanistically important roles in conferring anti-EGFR resistance in HNC as discussed above.

### Targeting cancer metabolism

Deregulating cellular energetics or reprogramming of cellular metabolism is an important emerging hallmark of cancer^88^. Normally, eukaryotic cells under aerobic conditions utilize glucose to produce pyruvate by glycolysis, after which the pyruvate then enters the tricarboxylic acid cycle in the mitochondria and consequently yields 36 molecules of ATP by OXPHOS. However, in anaerobic conditions, glycolysis is favored instead of OXPHOS and glucose is catabolized to lactate, which results in the generation of 2 molecules of ATP. Common features of the altered energy metabolism in cancer cells are increased glucose uptake and preference for glycolysis even in the presence of oxygen (hence the term ‘aerobic glycolysis’), which is known as the ‘Warburg effect’^89^. Biological advantages which cancer cells expect from this metabolic rewiring despite its rather inefficient, counterintuitive process are faster rate of ATP production, reduced generation of reactive oxygen species, stability of glycolytic fueling under hypoxic conditions which many tumors lie, and increased shunting of glycolytic intermediates into various biosynthetic pathways to accomplish nucleotide, amino acid, and lipid synthesis needed for active cell proliferation. Cancer cells therefore enhance glycolysis by various compensatory mechanisms such as increasing glucose uptake by upregulating glucose transporter GLUT1 and activation of oncogenes (*RAS, MYC*) or mutation of tumor suppressor genes (*TP53*)^88,90,91^. Clinical implications of the Warburg effect are the incorporation of the glucose analog ^18^F-fluorodeoxyglucose, which is used in cancer diagnostics in positron emission tomography^92^, and the administration of yet another glucose analog, 2-deoxyglucose (2DG), as a therapy against many cancers including HNC^93,94^.

More recently, there has been an accumulation of refuting evidences against the Warburg effect. Unlike the initial understanding that cancer cells possess defective OXPHOS (hence the predominance of glycolysis), it is now widely accepted that cancer cells have normally functioning mitochondria capable of OXPHOS^95,96^. Especially under metabolic stress conditions such as restricted glucose supply, cancer cells can generate ATP through mitochondrial oxidation of cellular fuels other than glucose, most typically glutamine and fatty acids^97,98^. Whatsmore, unlike the anabolic metabolism with glycolysis, OXPHOS is mainly promoted for catabolic metabolism in cancer cells undergoing EMT. There are reports that in solid tumors, when cancer cells lose ECM attachment or migrate for metastasis, the cells markedly decrease glucose uptake and undergo energetic stress. The cells depend on FAO instead of glycolysis to meet the high demand for ATP required for cell survival^97,99,100^. Tumors with a lipogenic phenotype are associated with disease aggressiveness, worse prognosis, chemoresistance, and protection of cells against oxidative stress^101,102^. Therefore, therapies that act against mitochondrial DNA or OXPHOS should be viable anti-cancer strategies. Previous studies reported a reduction in the tumorigenic potential of cancer cells by targeting the mitochondrial DNA^103,104^, and agents such as metformin (an OXPHOS inhibitor) and etomoxir (an FAO inhibitor) are already being investigated as promising therapeutic options. Numerous studies have been conducted and have demonstrated that metformin is a potentially effective agent in HNC^105^. Specific suppression of FAO by CPT-1 inhibition with etomoxir demonstrates antitumor effects in pancreatic ductal adenocarcinoma and restores sensitivity to a conventional chemotherapeutic agent, gemcitabine^106^. Its effect on HNC has yet to be studied, but positive results are anticipated. Since cancer cells adapt to glycolysis inhibition by utilizing alternative nutrients to increase OXPHOS, the combinatorial treatment strategy of using both a glycolytic inhibitor (2DG) and an OXPHOS inhibitor (metformin) rather than either agent alone, has been studied as an effective treatment because it exerts a synergistic therapeutic effect in the induction of cell death^103,107^.

**Fig. 3:**
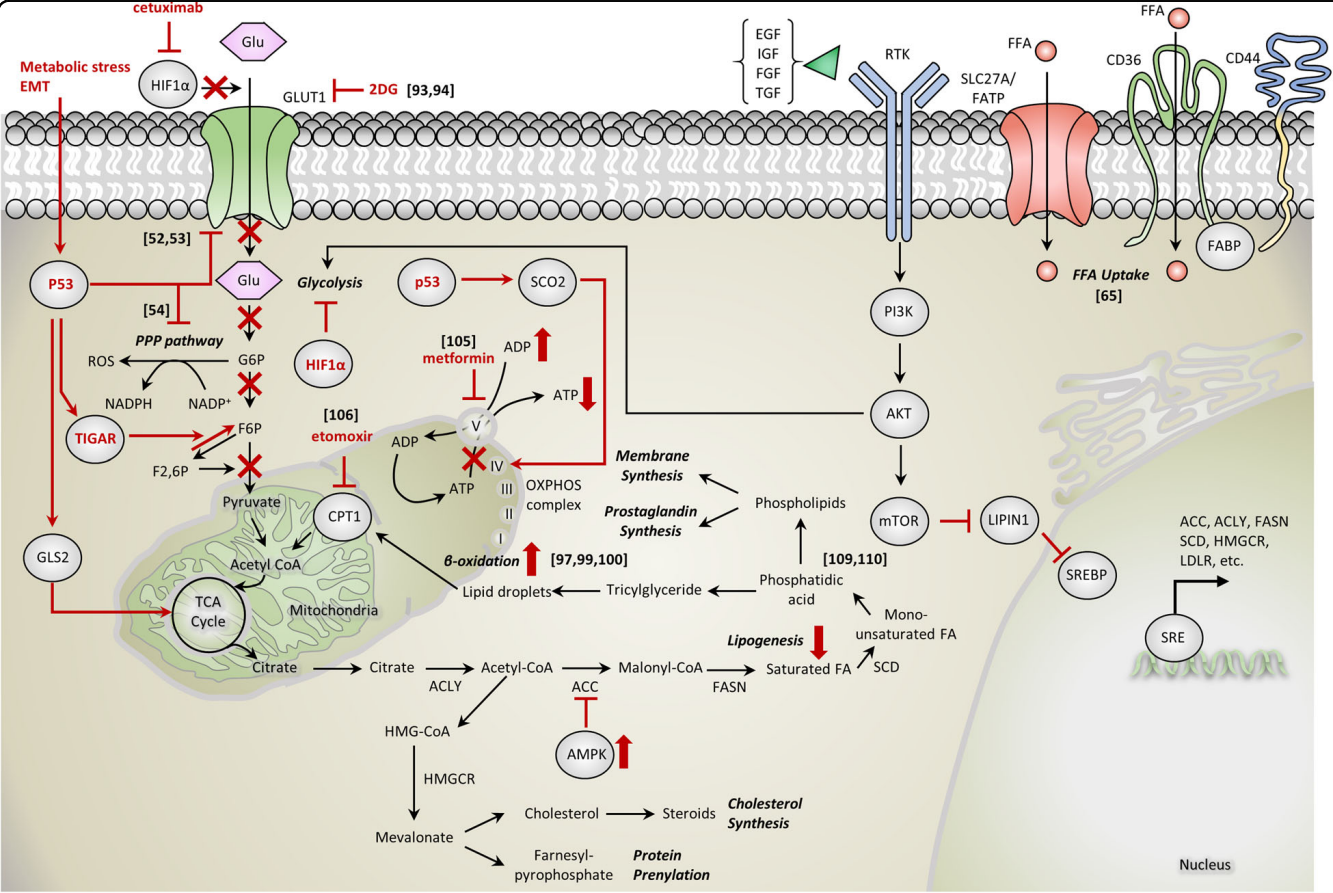
Metabolic patterns against bioenergetic stress in progressive HNC. Increased aerobic glycolysis or Warburg effect is a characteristic pattern of metabolism in cancers including HNC. However, due to metabolic stress, EMT changes, or certain drug treatments, cancer cells undergo metabolic rewiring where oxidative phosphorylation is favored instead of glycolysis. Specific actions and their consequences are highlighted in red. Additionally, possible connections between signaling pathways in cancer and cancer metabolism are suggested as shown. Numbers indicate relevant references in the text

Alteration in cellular energy metabolism is considered to be a universal hallmark of cancer. Increased glucose uptake, enhanced glycolysis and FAO are also distinctive hallmarks of HNC (Fig. 3)^108^. As previously discussed, mutant p53 plays many central roles in not only the initiation and progression of HNC but also in the context of cancer metabolism, where it modulates the glycolysis pathway in multiple ways. In addition, based on the association of CD44 and the initiation of metastasis of HNC cells by CD36-mediated fatty acid uptake, this HNC-specific FAO could be a potentially effective target by which metastasis and progression of HNC can be controlled^65^. This widespread but distinctive trait of cancer can be exploited as a therapeutic target and it is expected that it will complement the former targeted therapy by overcoming its current limitations. Recently, some have suggested a close relationship between oncogenic signaling such as the AKT-mTOR axis, which is the downstream signaling pathway of EGFR and other RTKs, and lipid metabolism, which is centered on oncogenic activation of sterol regulatory element-binding proteins^109,110^. A recent report has linked cetuximab resistance with the rewiring mechanism of cancer metabolism in HNC^111^. Cellular stress exerted by cetuximab reverses the Warburg effect, which in turn induces FAO stimulation and fatty acid synthesis inhibition through AMP-activated protein kinase activation. Therefore, future research should be directed toward this innovative approach to achieve novel insights and to advance HNC treatment. By simultaneously targeting two distinct areas of cancer, signaling networks and metabolism, the therapeutic effects would be maximized.

## Conclusion

In this article, studies concerning targeted therapies that involve central EGFR signaling in HNC have been comprehensively reviewed. Although numerous resistance mechanisms to EGFR-targeted therapies have been reported in HNC, EGFR is still important as an integral point for convergent signaling pathways, and therefore, EGFR targeting should form the basis of oncogenic signaling inhibition. As previously suggested, strategies that reinforce oncogenic signaling inhibition by dual inhibition of the ECD and the intracellular tyrosine kinase domain of EGFR or horizontal/vertical targeting should be considered.

**Fig. 4:**
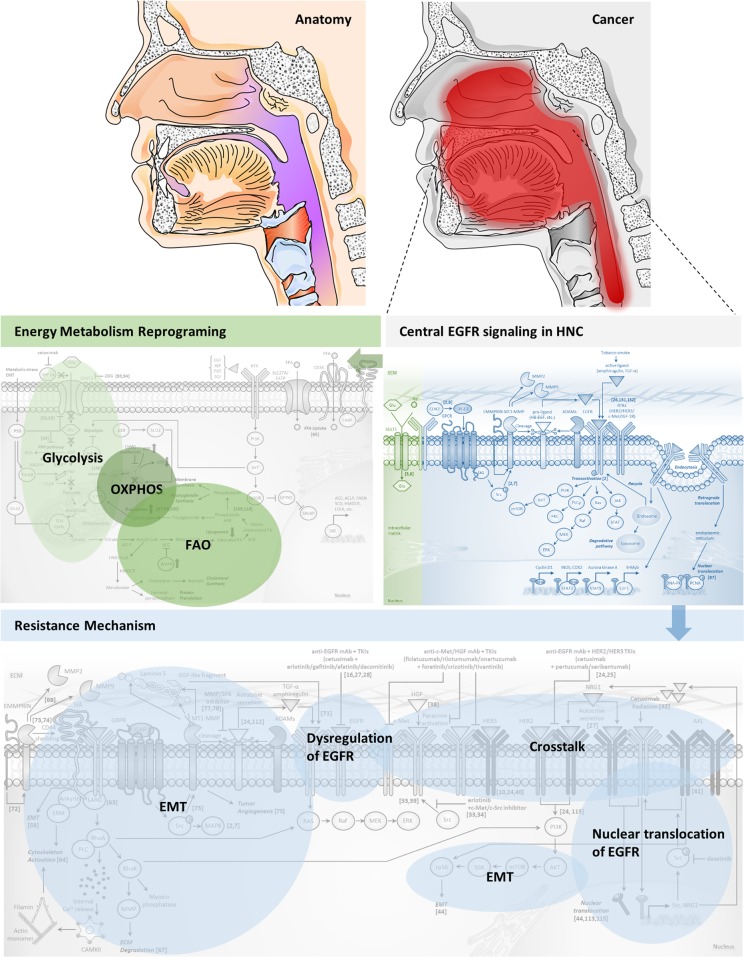
Multilateral treatment strategy based on EGFR signaling and cancer metabolism to stop the progression of HNC. From various resistance mechanisms against EGFR-targeted therapy and energy metabolism reprogramming in EGFR-centered HNC undergoing progression, the targeting of both EGFR central signaling and metabolism may be a rational treatment strategy

Herein, one notable characteristic feature of HNC is reprogrammed energy metabolism, which involves enhanced glycolysis and alternative activation of OXPHOS. Targeting cancer metabolism would be a distinctive strategy from targeting signaling pathways, and the combination of these two different approaches holds novel promise in exerting a multilateral combat strategy against HNC (Fig. 4). From several clues that suggest connections between cancer metabolism and signaling pathways in HNC, a more detailed understanding of how cancer metabolism is mechanistically involved in EGFR-mediated signaling in HNC is required. In the era of precision medicine and multiomics, evaluating the genetic profile of each individual patient and incorporating the patient profile into therapeutics form the basis of modern cancer treatment and preparing a multilateral strategy would be feasible for HNC patients in the next future.
